# Efficient selective removal of indole by Mg/Al-LDH@MOF: mechanistic insights and adsorption behavior

**DOI:** 10.1039/d6ra04492a

**Published:** 2026-07-31

**Authors:** Wei Zhang, Ding Peng, Aihe Wang, Hai Lin, Xuchao Yan, Zhihao Xu, Mingyou Yan, Zhenning Deng

**Affiliations:** a School of Municipal and Geomatics Engineering, Hunan City University Yiyang Hunan China hnweizhang@163.com hnwaihe409@163.com xuzhihao1102@163.com 3013155218@qq.com dengzn202@163.com; b School of Municipal and Environmental Engineering, Shenyang Jianzhu University Shenyang Liaoning China 18675701859@163.com; c Hunan Provincial Village Drinking Water Quality Safety Engineering Technology Research Center Yiyang Hunan China; d Yiyang City Commodity Quality Supervision and Inspection Institute Yiyang Hunan China 3085295989@qq.com 2476437713@qq.com

## Abstract

Due to the high toxicity and carcinogenicity of indole, wastewater should be treated to remove indole before discharge. In this study, a novel composite material, Mg/Al-LDH@MIL-101(Fe), was developed by integrating MIL-101(Fe), a high-porosity metal–organic framework (MOF), with Mg/Al-layered double hydroxide (LDH) nanosheets. This composite was designed for the efficient adsorption and removal of indole. The crystal structure, surface morphology, and chemical composition of the composite were analyzed using X-ray diffraction (XRD), Raman spectroscopy, X-ray photoelectron spectroscopy (XPS), and other characterization techniques. The results indicate that the composite exhibits a higher specific surface area and a greater number of active adsorption sites compared to Mg/Al-LDH and MIL-101(Fe). The adsorption of indole can be effectively removed by electrostatic adsorption on the surface of the material, and π–π stacking. The experimental results demonstrated that the indole removal rate could reach 93.14% under the following conditions: pH = 7, adsorbent dosage of 0.1 g L^−1^, adsorption time of 2 hours, and indole concentration of 20 mg L^−1^. With excellent recyclability (>82% efficiency after 5 cycles) and anti-interference capability (>86% efficiency under coexisting species), this work advances the potential for scalable coking wastewater treatment.

## Abstract 2

The Mg/Al-LDH@MIL-101(Fe) composite enables efficient adsorptive removal of indole through the synergistic effects of π–π interactions, metal coordination, and hydrogen bonding.

## Graphical abstract

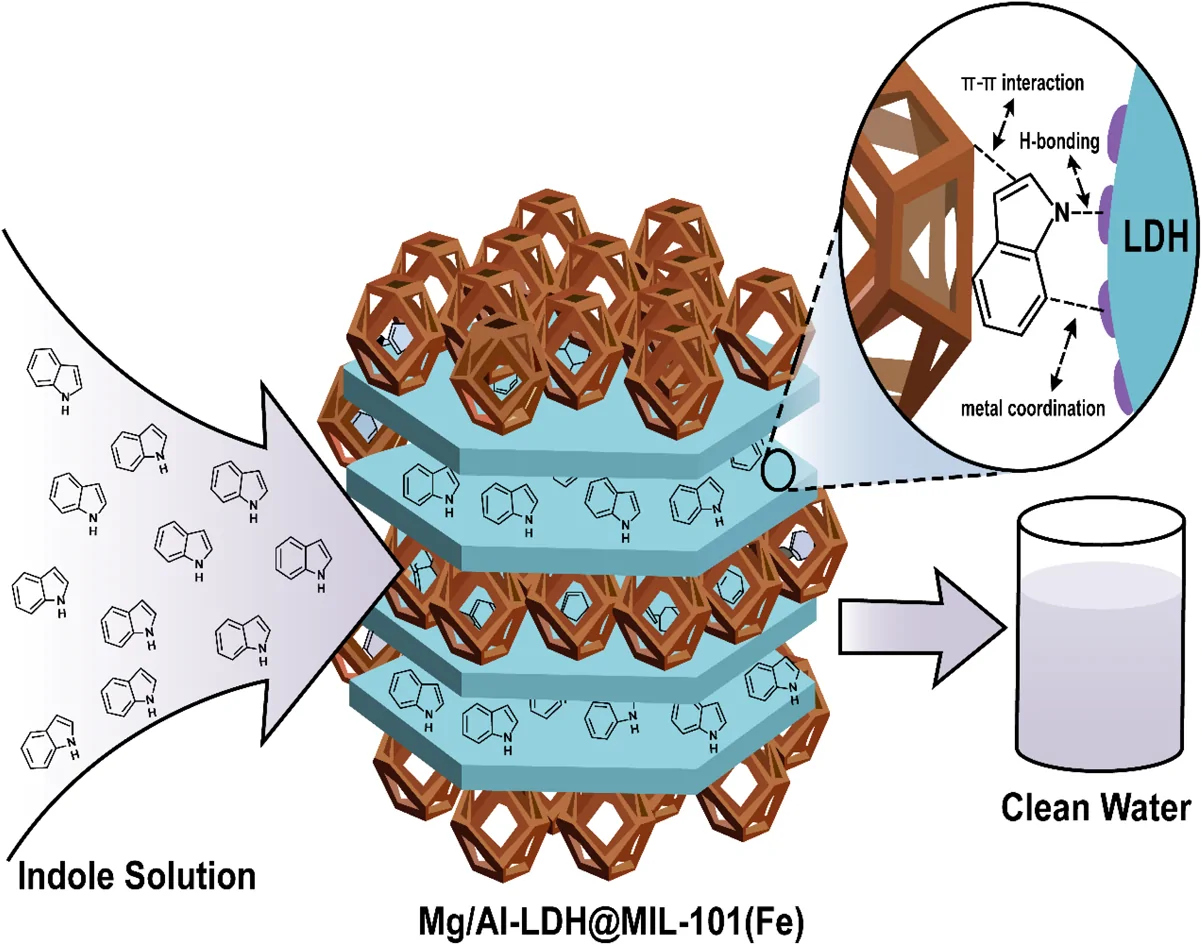

## 1. Introduction

Nitrogen-containing heterocyclic compounds present in coking wastewater are a class of highly toxic organic compounds that readily diffuse.^1^ Among these compounds, indole is a typical nitrogen-containing heterocyclic compound characterized by a stable ring structure, which renders it difficult to degrade and remove in natural environments.^2,3^ High concentrations of indole exert significant toxic effects on aquatic organisms and also pose a threat to human health.^4–9^ Therefore, the development of efficient and cost-effective indole removal technology has become an urgent priority in the field of water treatment.

Currently, the methods for wastewater treatment mainly include chemical precipitation,^10^ solvent extraction, membrane filtration,^11^ oxidation–reduction,^12^ and adsorption,^13^ among others. B. M. *et al.*^10^ employed chemical precipitation using lime (Ca(OH)_2_), caustic soda (NaOH), and soda ash (Na_2_CO_3_) to remove coexisting heavy metals (Cu(II) and Zn(II)) from cable industry wastewater. Their results demonstrated that the removal efficiency of heavy metals could reach over 90%. Among existing indole treatment technologies, the biological treatment method utilizes microorganisms or plants to treat indole without causing secondary chemical pollution. However, the biological treatment method exhibits a low tolerance threshold for highly toxic indoles and demonstrates poor resistance to impact loads;^14^ the extraction method exhibits high selectivity, and the chemical reaction proceeds rapidly. However, both the extraction method and the chemical method encounter technical challenges, including secondary pollution and extended treatment times.^15^ In recent years, the adsorption method has been extensively studied due to its simple operation, low energy consumption, and broad adaptability.^16^

LDH is an environmentally friendly nano-adsorption material that is widely utilized for pollutant adsorption and removal. This is attributed to its low cost, high efficiency, anion exchange capacity, and adjustable internal structure.^17^ However, the dense stacking structure of LDH limits its inherent adsorption capacity,^18^ as it reduces the number of active sites and mass transfer efficiency. Therefore, preventing the spontaneous stacking and aggregation of LDH is critical.

As a typical three-dimensional ordered mesoporous crystal within the MOF family, MIL-101(Fe) exhibits excellent chemical stability and a high specific surface area.^19^ These properties enable it to effectively inhibit the stacking of LDH nanosheets, making it an ideal candidate for constructing LDH composites. Pan *et al.*^20^ combined MIL-101(Fe) with Mg/Fe-LDH to remove phosphate. The results indicated that the composite material demonstrated superior adsorption performance compared to individual adsorbents. Currently, numerous studies have been conducted on Mg/Al-LDH and MIL-101(Fe) as individual materials. However, there is limited research on the Mg/Al-LDH@MIL-101(Fe) composite adsorbent formed by combining these two materials, particularly regarding their adsorption and desorption efficiency for indole. It is necessary to clarify the detailed changes in the structural and adsorption characteristics of the composite material, and further elucidate the adsorption mechanism of the composite material for indole.

In this study, Mg/Al-LDH@MIL-101(Fe) was synthesized using the solvothermal method. The obtained composites were characterized and analyzed, and their adsorption properties for indole were evaluated. Furthermore, the effects of material ratio, reaction temperature, and reaction time on the indole removal capacity were investigated to determine the optimal preparation conditions.Concurrently, the effects of multiple factors—including pH, adsorbent dosage, adsorption time, indole concentration, and coexisting ions—on the adsorption removal process were investigated. Adsorption performance and mechanism were explored through adsorption kinetics, thermodynamics studies, relevant characterization techniques, and desorption cycle experiments. The objective of this study is to provide a novel approach for the development of efficient adsorbents and the advancement of coking wastewater treatment.

## 2. Materials and methods

### 2.1 Instruments and reagents

Experimental instruments: UV-1800 UV-Visible Spectrophotometer; BSA124S-CW Electronic Balance; LLS-20-L Ultrapure Water Machine; HJ-6A Constant Temperature Magnetic Stirrer; DHG-9030A Electric Heating Constant Temperature Blast Drying Oven; DZ-1BCII Vacuum Drying Oven; PHSJ-5 pH Meter; GJ-2 Sealed Sample Preparation and Crushing Machine.

Experimental materials: indole (purity ≥99%), cobalt nitrate hexahydrate, aluminum nitrate nonahydrate, urea, ammonium fluoride, ferric chloride hexahydrate, phthalic acid, and polyvinylpyrrolidone were all of analytical grade. The experimental water used was ultrapure.

### 2.2 Material preparation

#### 2.2.1 Preparation of Mg/Al-LDH

A mixed salt solution was prepared by dissolving 3.845 g of Mg(NO_3_)_2_·6H_2_O and 1.875 g of Al(NO_3_)_3_·9H_2_O in 50 mL of deionized water. Subsequently, 3 g of urea was added, and the mixture was stirred at room temperature for 5 minutes to ensure complete dissolution. The resulting clarified solution was transferred to a 100 mL PTFE-lined high-pressure reactor and crystallized at 110 °C for 24 hours. Upon completion of the reaction, the system was cooled naturally. The product was centrifuged, washed three times with deionized water, and finally dried at 60 °C for 24 hours to obtain the Mg/Al-LDH sample.

#### 2.2.2 Preparation of MIL-101(Fe)

Dissolve 0.332 g of terephthalic acid and 1.081 g of ferric chloride hexahydrate in 30 mL of *N*,*N*-dimethylformamide (DMF) and stir at room temperature for 30 minutes until a homogeneous solution is obtained. Subsequently, transfer the resulting clear solution to a 100 mL PTFE-lined high-pressure reactor and crystallize at 110 °C for 12 hours. After completion of the reaction, allow the system to cool naturally to room temperature. The resulting orange precipitate is then centrifuged and washed three times with DMF and anhydrous ethanol to remove unreacted raw materials and residual solvents. Finally, obtain the MIL-101(Fe) sample by drying the product in a blast drying oven at 60 °C for 6 hours.

#### 2.2.3 Preparation of Mg/Al-LDH@MIL-101(Fe)

0.1 g of Mg/Al-LDH was dissolved in 60 mL of DMF, followed by the sequential addition of 0.5405 g of FeCl_3_·6H_2_O, 0.166 g of H_2_BDC, and 0.3 g of polyvinylpyrrolidone. After stirring for 1 hour, the resulting solution was transferred to a polytetrafluoroethylene-lined autoclave and reacted at 110 °C for 24 hours to yield the MgAl-LDH@MIL-101(Fe) composite.

### 2.3 Characterization methods

The morphology of the samples was observed by scanning electron microscopy (SEM, GeminiSEM 300). The crystal structure was characterized by X-ray diffraction (XRD, Rigaku SmartLab 9). The surface functional groups were analyzed by Fourier-transform infrared spectroscopy (FTIR, Thermo Scientific Nicolet iS50). The elemental composition was determined by X-ray photoelectron spectroscopy (XPS, Thermo Scientific K-Alpha). The specific surface area, pore volume, and pore size distribution were measured using a fully automatic surface area and pore size analyzer (BET, Autosorb-iQ).

### 2.4 Test method

#### 2.4.1 Response surface optimization of composite material preparation conditions

The effects of hydrothermal temperature, hydrothermal time, and the ratio of MIL-101(Fe) to Mg/Al-LDH on the adsorption performance of indole were investigated through single-factor experiments. Based on the results of these single-factor experiments, a response surface model was constructed using Box–Behnken design to optimize the preparation process of the composite photoadsorbent. The resulting optimal adsorbent will be employed for subsequent performance studies.

#### 2.4.2 Adsorption test

The effects of pH, adsorbent dosage, adsorption time, initial indole concentration, and coexisting ions on the removal rate were systematically investigated by adding Mg/Al-LDH, MIL-101(Fe), and Mg/Al-LDH@MIL-101(Fe) adsorbents to indole simulated wastewater. After filtering a 5 mL sample through a 0.22 µm filter membrane, absorbance was measured at 280 nm, and the removal rate was calculated according to eqn (1).1
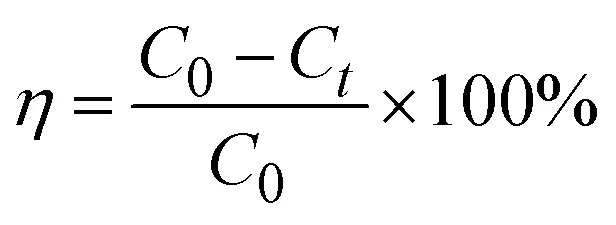
In the equation, *η* represents the removal rate of indole (%); *C*_0_ and *C*_*t*_ (mg L^−1^) are the initial concentration of indole and the instantaneous concentration at time *t* (min), respectively.

#### 2.4.3 Adsorption kinetics and isothermal models

Adsorption kinetics: the adsorption process was analyzed and fitted using quasi-first-order and quasi-second-order kinetic models, as presented in eqn (2) and (3).

2 ln(*q*_e_ − *q*_*t*_) = −*k*_1_*t* + ln *q*_e_

3
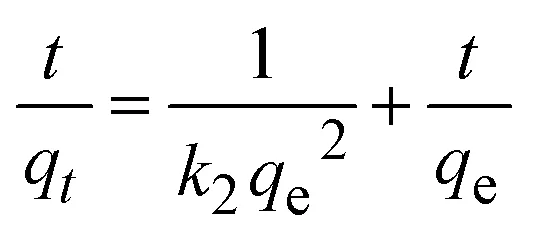
In the equation, *q*_*t*_ (mg g^−1^) represents the adsorption amount at time *t*; *q*_e_ (mg g^−1^) is the equilibrium adsorption capacity; *k*_1_ (min^−1^) and *k*_2_ (g mg^−1^ min^−1^) are pseudo-first-order and pseudo-second-order adsorption rate constants, respectively.

Adsorption isotherms: Langmuir and the Freundlich isotherm models were used to describe the equilibrium data, as shown in eqn (4) and (5).4
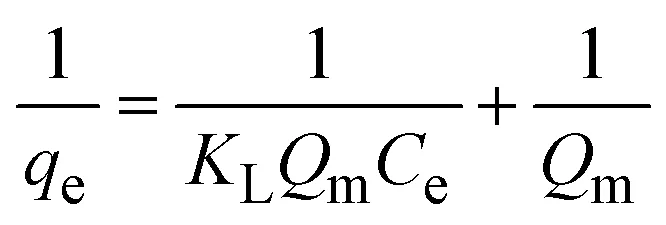
5
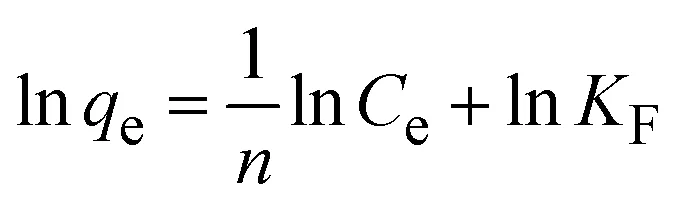
In the equation, *q*_e_ and *Q*_m_ (mg g^−1^) are the equilibrium adsorption capacity and the theoretical maximum adsorption capacity, respectively. *C*_e_ (mg L^−1^) is the equilibrium concentration of adsorbate; *K*_L_ (mg L^−1^) and *K*_F_ (mg L^−1^) are the adsorption equilibrium constants of Langmuir and the Freundlich models, respectively. *n* is a constant that characterizes the strength of adsorption.

## 3. Results and discussion

### 3.1 Characterization of Mg/Al-LDH, MIL-101(Fe) and Mg/Al-LDH@MIL-101(Fe)

**Fig. 1:**
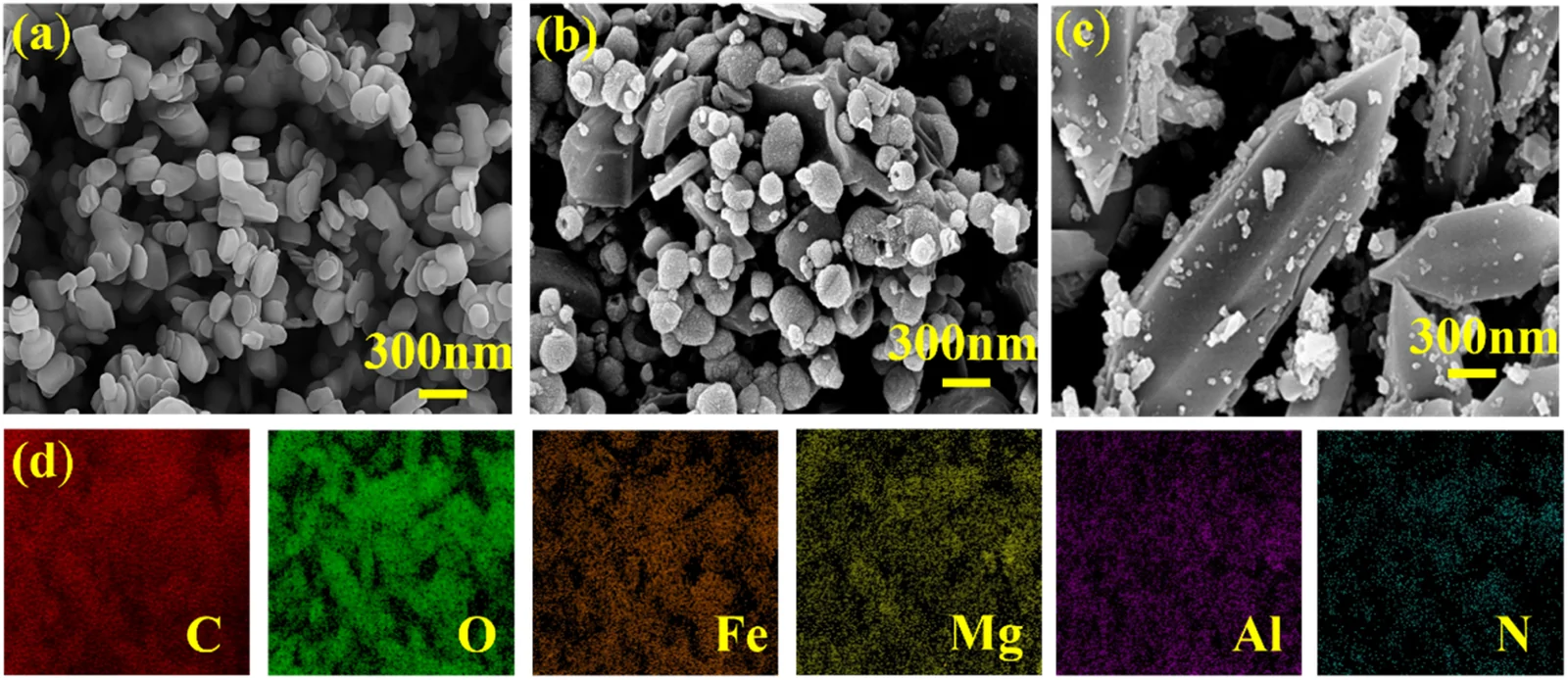
SEM images of (a) Mg/Al-LDH, (b) MIL-101 (Fe), and (c) Mg/Al-LDH@MOF. EDS images of (d) Mg/Al-LDH@MOF.

The microstructures of Mg/Al-LDH, MIL-101(Fe), and Mg/Al-LDH@MOF were illustrated in Fig. 1 and S1. Mg/Al-LDH exhibits a typical layered hydrotalcite structure (Fig. 1a, S1a and b). The MIL-101(Fe) sample shows a clear and uniform octahedral morphology (Fig. 1b, S1c and d). SEM images of Mg/Al-LDH@MOF composites (Fig. 1c, S1e and f) clearly reveal that Mg/Al-LDH nanosheets are tightly combined with MIL-101(Fe) octahedrons to form heterostructures. It is worth noting that compared with the original stacked Mg/Al-LDH, the Mg/Al-LDH substrate in the composite exhibits a more dispersed morphology with an increased apparent size. This indicates that the addition of MIL-101(Fe) effectively inhibits the restacking of Mg/Al-LDH nanosheets, thereby providing greater room for expansion. Concurrently, the size of MIL-101(Fe) crystals grown *in situ* on the surface is smaller than that of MIL-101(Fe) synthesized directly. This is attributed to the fact that Mg/Al-LDH provides a greater number of active sites for MIL-101(Fe) nucleation, leading to the formation of a large quantity of MIL-101(Fe) nuclei and restricted growth space. The presence of characteristic elements from the two components in the EDS spectrum (Fig. 1d) provides strong evidence for the successful preparation of the composite material.

**Fig. 2:**
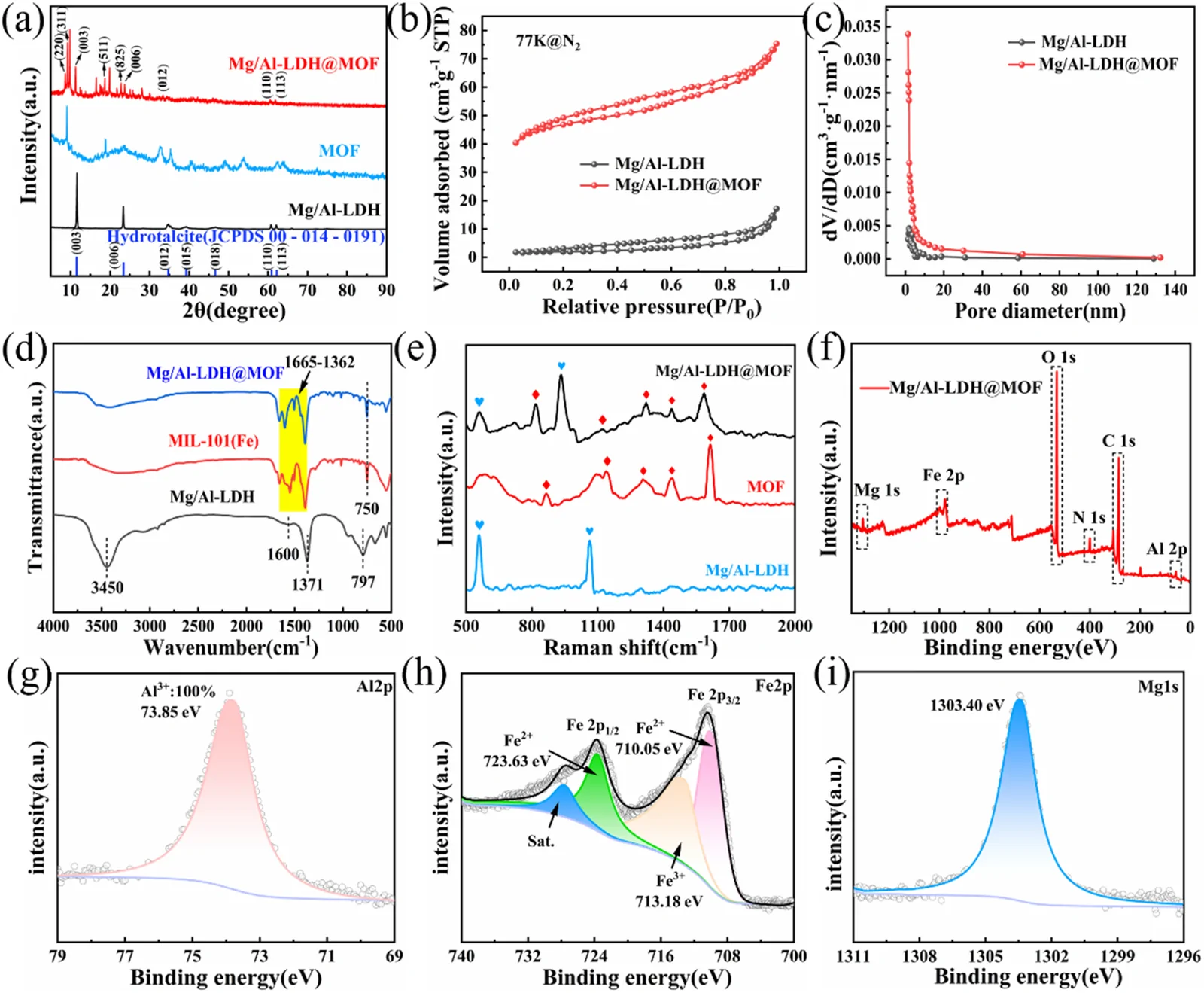
(a) XRD patterns of Mg/Al-LDH, MIL-101(Fe), and Mg/Al-LDH@MOF. (b) N_2_ adsorption–desorption isotherms and (c) pore size distribution of Mg/Al-LDH and Mg/Al-LDH@MOF. (d) FT-IR spectra, and (e) Raman spectra of Mg/Al-LDH, MIL-101(Fe), and Mg/Al-LDH@MOF. XPS spectra of Mg/Al-LDH@MOF: (f) survey, (g) Al 2p, (h) Fe 2p, (i) Mg 1s.

The crystal phase composition of Mg/Al-LDH, MIL-101(Fe) and Mg/Al-LDH@MOF was characterized by X-ray diffraction (XRD) (Fig. 2a). A series of characteristic diffraction peaks, such as (003) and (006), appeared at lower angles in Mg/Al-LDH samples, confirming the successful formation of the typical layered double hydroxide crystal structure.^21^ The diffraction peaks observed for MIL-101(Fe) at 2*θ* values of 10.17°, 17.94°, and 22.91° correspond to the (311), (511), and (825) crystal planes, respectively.^22^ The XRD pattern of Mg/Al-LDH@MOF exhibits both the characteristic peaks corresponding to the layered structure of Mg/Al-LDH and the diffraction signals from the MIL-101(Fe) component. This result strongly confirms that the Mg/Al-LDH@MOF hybrid material has been successfully synthesized *via* the composite process, with the process preserving the original crystal structural integrity of each constituent component.

The pore structures of Mg/Al-LDH and Mg/Al-LDH@MOF were characterized by BET. As illustrated in Fig. 2b, both samples exhibit type-IV isotherms with distinct H3 hysteresis loops, indicating that the pore structure of the material is predominantly mesoporous with a morphology primarily consisting of slit-shaped pores.^23,24^ Compared with Mg/Al-LDH, the Mg/Al-LDH@MOF composites show significantly higher nitrogen adsorption capacity across the entire relative pressure range, indicating a substantial increase in its specific surface area and total pore volume. The pore size distribution was obtained using the NLDFT calculation model. The corresponding pore size distribution plot reveals that the composite material possesses a broader pore size distribution, with additional pores appearing in the larger mesoporous scale (Fig. 2c). This hierarchical porous structure, which integrates the inherent pores of the two components and newly formed interparticle voids, is conducive to providing abundant active sites and promoting mass transfer. This is expected to enhance the material's performance in adsorption applications.

The functional groups of Mg/Al-LDH, MIL-101(Fe), and Mg/Al-LDH@MIL-101(Fe) were investigated *via* FT-IR spectroscopy. As shown in Fig. 2d, the vibrational bands of Mg/Al-LDH at approximately 3450 cm^−1^ and 1600 cm^−1^ correspond to O–H stretching vibration^25,26^ and interlayer water molecule bending vibration,^27,28^ respectively. The peak at 1371 cm^−1^ is attributed to C–O bond vibration, and the absorption peak at 797 cm^−1^ is related to metal–oxygen (M–O) or metal–hydroxide (M–OH) vibration.^29^ In MIL-101(Fe), the two peaks between 1665 and 1362 cm^−1^ represent the symmetric and asymmetric vibrations of the carboxylate C–O bond, respectively.^30,31^ The C–H out-of-plane bending vibration at 750 cm^−1^ corresponds to the benzene ring of terephthalic acid. The absorption spectra of Mg/Al-LDH@MOF composites were nearly consistent with those of MIL-101(Fe) and Mg/Al-LDH, indicating that the precursor structure was retained following compounding. This confirms the successful preparation of the composites.

The structure of Mg/Al-LDH, MIL-101(Fe), and Mg/Al-LDH@MOF were analyzed using Raman spectroscopy. As shown in Fig. 2e, the vibration bands of MIL-101(Fe) appear at 862, 1132, 1306, 1429 and 1608 cm^−1^, indicating the presence of aromatic rings and dicarboxylic acid groups.^32^ The band observed in the 557 cm^−1^ region of Mg/Al-LDH is characteristic of the octahedral structure of the hydrotalcite-like layer, corresponding to the stretching vibration of the Me–O–Me bond. The band at 1063 cm^−1^ arises from the vibration of intercalated carbonate anions.^33^ Notably, the peak at approximately 800 cm^−1^ exhibited a shift after the formation of the composite, which may be attributed to chemical interactions between Mg/Al-LDH and MIL-101(Fe).^34^ The abundant hydroxyl groups on the LDH surface may form weak hydrogen bonds or coordination interactions with the MIL-101(Fe) framework, thereby perturbing the electronic environment of the aromatic ring and the dicarboxylate group vibrations.

The composition and chemical state of the adsorbents were investigated using X-ray photoelectron spectroscopy (XPS). Characteristic peaks corresponding to C 1s, O 1s, Fe 2p, Mg 1s, Al 2p, and N 1s were detected in Fig. 2f, confirming the presence of C, O, Fe, Mg, Al, and N elements in Mg/Al-LDH@MIL-101(Fe). The C 1s XPS spectrum of MgAl-LDH@MIL-101(Fe) exhibits three peaks at 288.75 eV, 286.35 eV, and 284.80 eV, corresponding to O=C–O, C–O, and C–C bonds, respectively (Fig. S2a).^35,36^ The O 1s spectrum displays a prominent peak at 530.84 eV, which is primarily attributed to the presence of M–O bonds in the material (Fig. S2b),^37,38^ while the peak at 531.33 eV is assigned to hydroxyl (–OH). As shown in Fig. S2c, in the N 1s spectrum shows a peak at 399.58 eV arising from an amide bond, where the nitrogen atom of the amide bond is bonded to a carbon atom *via* a single bond. Peaks at 400.75 eV and 398.56 eV are associated with NH–C and N–C bonds, respectively.^39,40^ The Al 2p spectrum (Fig. 2g) exhibits a single prominent peak, which is attributed to the presence of Al–O and Al–OH in LDH.^41^ The Fe 2p spectrum is decomposed into 710.05, 713.18, 723.63, and 727.63 eV, corresponding to Fe 2p_3/2_ (Fe^2+^), Fe 2p_3/2_ (Fe^3+^), Fe 2p_1/2_ (Fe^2+^), and Fe^2+^ satellite peaks, respectively (Fig. 2h).^42–44^ In the Mg 1s spectrum (Fig. 2i), a peak corresponding to the Mg–O bond is observed at 1303.40 eV.^45^

### 3.2 Optimum preparation conditions of Mg/Al-LDH@MIL-101(Fe)

To evaluate the effect of preparation conditions on the adsorption performance of Mg/Al-LDH@MIL-101(Fe) and further optimize the adsorbent preparation conditions, the response surface methodology design principle was followed.^46^ Hydrothermal temperature, the ratio of loading material Mg/Al-LDH to MIL-101(Fe), and hydrothermal time were selected as independent variables, with indole removal rate as the response value, to establish a model. The Box–Behnken response surface method was employed to optimize these three factors.

**Fig. 3:**
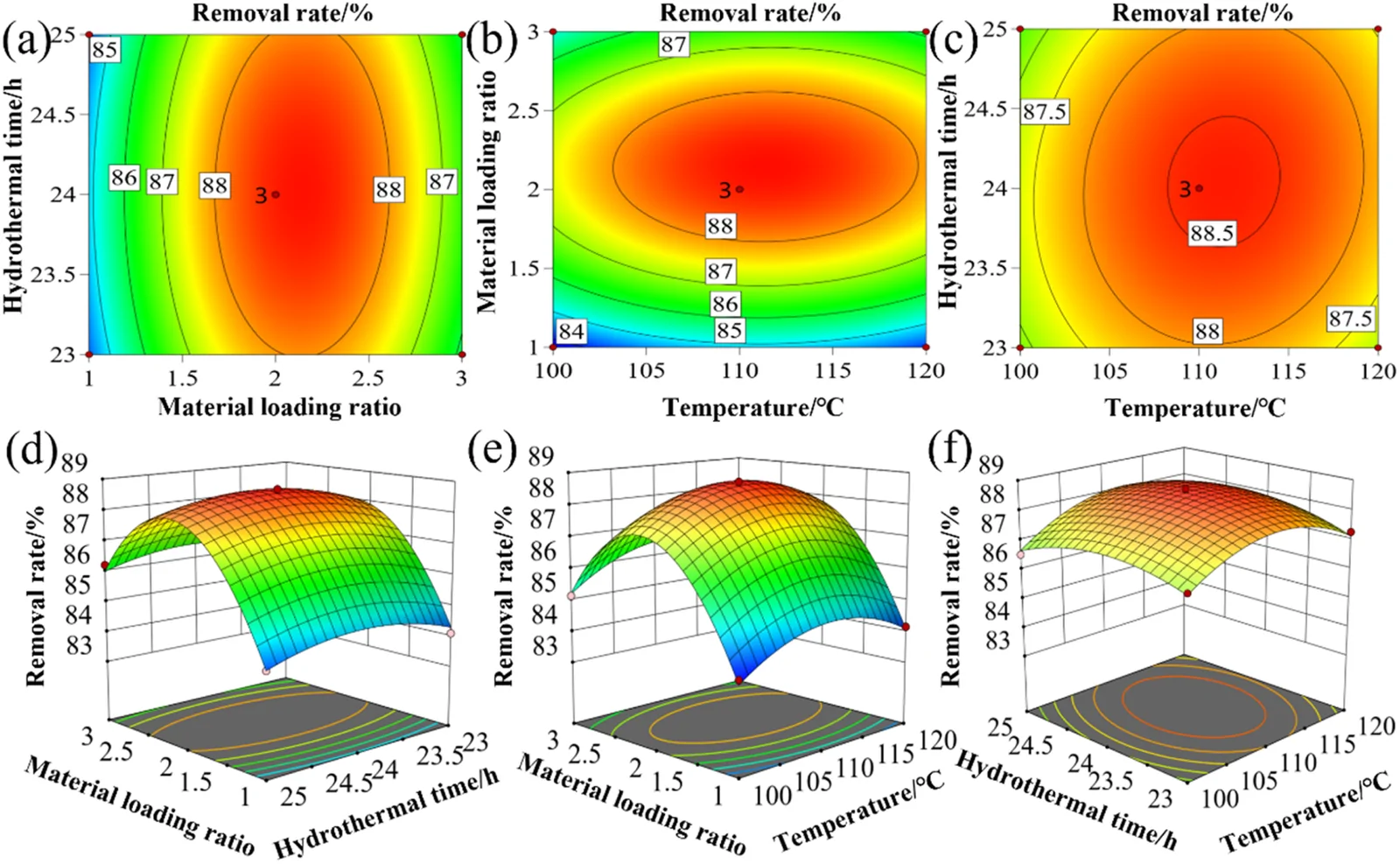
Contour plots of the response surface showing the interactive effects of: (a) composite material loading ratio *vs.* hydrothermal time, (b) composite material loading ratio *vs.* hydrothermal temperature, and (c) hydrothermal temperature *vs.* hydrothermal time. 3D surface plots of the response surface showing the interactive effects of: (d) composite material loading ratio *vs.* hydrothermal time, (e) composite material loading ratio *vs.* hydrothermal temperature, and (f) hydrothermal temperature *vs.* hydrothermal time.

As illustrated in Fig. 3a and b, the contour formed by the ratio of the loading material and hydrothermal time, as well as the contour formed by hydrothermal temperature and the ratio of the loading material, is elliptical. This indicates a significant interaction between these two groups of factors. Additionally, the contour map of hydrothermal temperature and hydrothermal time is approximately elliptical as shown in Fig. 3c, there is a certain degree of interaction between these two variables. Under this interaction, the indole removal rate was higher when the loading material ratio ranged from 1.5 to 2.5, the hydrothermal temperature ranged from 105 °C to 115 °C, and the hydrothermal time ranged from 23.5 hours to 24.5 hours. Fig. 3d and e indicate that the curvature of the loading material ratio is greater than that of hydrothermal temperature and time within the experimental range, and its effect on the indole removal rate is also more significant. The hydrothermal time and hydrothermal temperature surfaces exhibit relatively flat profiles with minimal curvature, indicating that these two factors have a limited influence on the indole removal rate (Fig. 3f). From the above analysis, the primary and secondary order of influence of the three preparation factors on the indole removal rate is determined as follows: loading material ratio > hydrothermal temperature > hydrothermal time. Under the conditions of a loading material ratio of 1 : 2, a hydrothermal temperature of 110 °C, and a hydrothermal time of 24 hours, the indole removal rate reached its maximum value of 88.57%.

### 3.3 Adsorption of indole

#### 3.3.1 Dynamics research

**Fig. 4:**
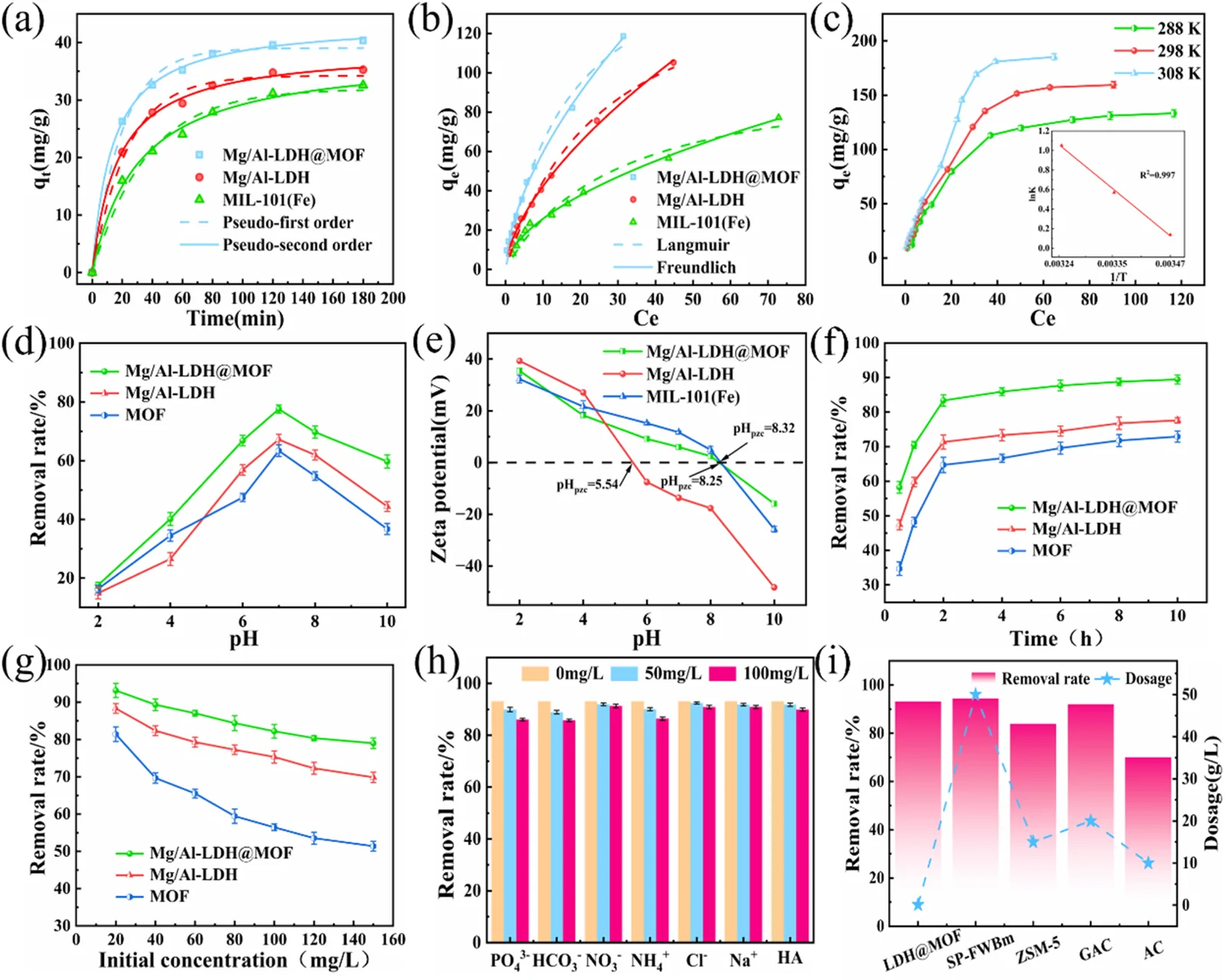
(a) Adsorption kinetics and (b) adsorption isotherms of indole on Mg/Al-LDH, MIL-101(Fe), and Mg/Al-LDH@MOF. (c) Equilibrium adsorption isotherms of indole onto Mg/Al-LDH@MIL-101(Fe) at different temperatures (Inset: van't Hoff plot). Effects of (d) pH (dosage of Mg/Al-LDH@MOF = 0.08 g L^−1^, adsorption time = 1 h, indole concentration = 50 mg L^−1^, *T* = 25 °C), (e) zeta potential, (f) adsorption time (pH = 7, dosage of Mg/Al-LDH@MOF = 0.1 g L^−1^, indole concentration = 50 mg L^−1^, *T* = 25 °C), (g) indole concentration (pH = 7, dosage of Mg/Al-LDH@MOF = 0.1 g L^−1^, adsorption time = 2 h, *T* = 25 °C), effects of (h) coexisting ions (pH = 7, dosage of Mg/Al-LDH@MOF = 0.1 g L^−1^, adsorption time = 2 h, indole concentration = 20 mg L^−1^, *T* = 25 °C) on the removal of indole by Mg/Al-LDH@MOF. (i) Comparison of indole removal efficiency and adsorbent dosage with previously reported adsorbents.

The pseudo-first-order and pseudo-second-order kinetic models were employed to fit the kinetic experimental data of indole adsorption onto Mg/Al-LDH, MIL-101(Fe), and Mg/Al-LDH@MOF, aiming to analyze the adsorption kinetic behavior of indole. As shown in Fig. 4a, indole was significantly removed during the initial 60 min for all three adsorbents, owing to the abundant active sites available on the sample surfaces. As these active sites gradually became occupied, the removal rate of indole decreased, and the adsorption process eventually reached equilibrium. Notably, the Mg/Al-LDH@MOF composite consistently exhibited higher adsorption capacity and faster adsorption rate than the single components throughout the entire adsorption process, confirming that the combination of Mg/Al-LDH and MIL-101(Fe) enhanced the adsorption performance. According to Table S2, the correlation coefficient (*R*^2^) of the pseudo-second-order kinetic model was superior to that of the pseudo-first-order model, revealing that chemisorption was the decisive step in the adsorption process.^47^

#### 3.3.2 Isothermal study

As shown in Fig. 4b, the adsorption properties of the samples were investigated by indole adsorption isotherms, with the data fitted to both the Langmuir and the Freundlich models. As indicated in Table S2, the Freundlich model (*R*^2^ values of 0.997, 0.997, and 0.994) provides a better description of the indole adsorption isotherm than the Langmuir model (*R*^2^ values of 0.978, 0.986, and 0.964). These results suggest that indole undergoes multilayer adsorption on the uniform surfaces of Mg/Al-LDH, MIL-101(Fe), and Mg/Al-LDH@MIL-101(Fe).^48^ Concurrently, the *n* values of the three materials in the Freundlich model are all greater than 1, indicating that the adsorption process of indole onto the three adsorbents is favorable.^49^ Based on the results of the Langmuir model, the maximum adsorption capacity (*q*_m_) of Mg/Al-LDH@MIL-101(Fe) is significantly higher at 175.732 mg g^−1^ compared to those of LDH (144.120 mg g^−1^) and MIL-101(Fe) (102.563 mg g^−1^). The adsorption isotherms of indole onto the Mg/Al-LDH@MIL-101(Fe) composite at different temperatures are presented in Fig. 4c. As the temperature increased from 288 K to 298 K and 308 K, the adsorption capacity of indole gradually increased, indicating that the adsorption process is endothermic in nature. The corresponding thermodynamic parameters are summarized in Table S3. The adsorption data were fitted using the van't Hoff equation, yielding a correlation coefficient (*R*^2^) of 0.997. Over the entire temperature range studied, the Gibbs free energy change (Δ*G*^*θ*^) remained negative, confirming that the adsorption of indole onto Mg/Al-LDH@MIL-101(Fe) is a spontaneous process. Furthermore, the absolute value of Δ*G*^*θ*^ increased with rising temperature, suggesting that higher temperatures are favorable for the adsorption process. In addition, the positive values of enthalpy change (Δ*H*) and entropy change (Δ*S*) indicate that the adsorption is endothermic and that the degree of disorder at the adsorbent–solution interface increases after adsorption.

#### 3.3.3 Analysis of influencing factors

The initial pH value of the solution may alter the surface charge of the adsorbent, thereby affecting the electrostatic interaction between the adsorbent and the contaminant. As shown in Fig. 4d, within the pH range of 2–10, the indole removal efficiency by the three adsorbent exhibited an increasing trend followed by a decreasing trend, reaching its maximum at pH 7, which was selected as the optimal pH value for the experiment. The lowest removal rate was observed at pH 2, attributed to the strong electrostatic repulsion between the positively charged adsorbent surface (pH < pHpzc, Fig. 4e) and the protonated indole species under highly acidic conditions. Furthermore, the strongly acidic environment may degrade the adsorbent material, further reducing the number of effective adsorption sites. MIL-101(Fe) demonstrated higher adsorption efficiency than Mg/Al-LDH at pH 4, which may be attributed to the 100-fold reduction in H^+^ concentration compared to pH 2. At this pH, most indole exists in the form of neutral molecules, which results in significantly weakened electrostatic repulsion, thereby enabling the MIL-101(Fe) material to leverage its π–π stacking interactions and substantial specific surface area advantage. Nevertheless, within a broader pH 6–8, Mg/Al-LDH exhibits good adsorption performance due to the hydrogen bonding of surface hydroxyl groups and metal bridging effects. The Mg/Al-LDH@MIL-101(Fe) composite successfully integrates the benefits of both materials and achieves the highest removal rate across all tested pH conditions.

Fig. S3 illustrates the effect of dosage of Mg/Al-LDH, MIL-101(Fe), and Mg/Al-LDH@MIL-101(Fe) on the removal efficiency of indole. Within the dosage range of 0.04–0.1 g L^−1^, the indole removal rates increased from 57.64%, 53.41%, and 47.58% to 86.34%, 76.21%, and 68.59%, respectively, as the adsorbent dosage increased. This is attributed to the higher adsorbent dose providing more active sites for indole adsorption, thereby facilitating rapid indole removal. With further increases in adsorbent dosage, the adsorption rate changes became insignificant. This is because excessive adsorbent particles may aggregate, obscuring some internal active sites and preventing them from contacting pollutants, which results in a decrease in the adsorption efficiency per unit mass of adsorbent. Therefore, 0.1 g L^−1^ was selected as the optimal adsorbent dosage for the experiment.

Fig. 4f investigates the effect of adsorption time on indole removal by various adsorbents. The results indicate that during the initial stage (approximately 2 hours), the adsorption rate is faster due to a higher concentration gradient and an abundance of surface active sites. Subsequently, as the driving force diminishes and surface sites approach saturation, the adsorption rate gradually decreases until equilibrium is reached. Therefore, 2 hours is determined to be the optimal reaction time for the experiment. Additionally, the Mg/Al-LDH@MIL-101(Fe) composite exhibited the highest adsorption performance, with its equilibrium adsorption capacity significantly superior to that of individual components. This is primarily attributed to the effective integration of the structural characteristics of Mg/Al-LDH and MIL-101(Fe) to form a unique structure featuring multi-level pores and a rich array of active sites, along with a synergistic effect between the two components, thereby enhancing the adsorption capacity for indole.

Within the concentration range of 20–150 mg L^−1^, indole concentration was employed to examine the effect of initial concentration on removal efficiency. As illustrated in Fig. 4g, the removal rate decreased from 93.14%, 88.13%, and 79.81% to 79.16%, 70.14%, and 51.44% as the indole concentration increased from 20 mg L^−1^ to 100 mg L^−1^ following a 2-hour adsorption period. This phenomenon can be attributed to the saturation of effective active sites and the intensification of competitive adsorption between substrates and adsorbents. Under high concentration conditions, the limited active sites are unable to fully contact and remove additional indole molecules, resulting in a decline in removal efficiency. The concentration of indole in coal chemical wastewater typically ranges from 10 mg L^−1^ to 20 mg L^−1^,^50^ therefore 20 mg L^−1^ was identified as the optimal reaction concentration in this experiment.

In actual wastewater, indole frequently coexists with a variety of inorganic anions, cations, nitrogen-containing species, and other organic matter, which may influence its adsorption process. To evaluate the selectivity of the prepared composites, interference experiments were conducted using six typical ions (PO_4_^3−^, HCO_3_^−^, NO_3_^−^, NH_4_^+^, Cl^−^, and Na^+^) and organic humic acid (HA), with different concentration groups and a blank control established (Fig. 4h). The results indicated that as the ionic strength of HCO_3_^−^ and NH_4_^+^ increased, the adsorption rate of indole decreased gradually. This was primarily attributed to the hydrolysis of HCO_3_^−^ and NH_4_^+^, which altered the solution pH and inhibited indole adsorption. When the PO_4_^3−^ concentration increased from 0 mg L^−1^ to 100 mg L^−1^, the adsorption rate decreased from 93.14% to 86.63%. This decline may be attributed to the higher charge and larger hydrated radius of PO_4_^3−^, enabling it to undergo ion exchange with the primary interlayer anions (CO_3_^2−^) in Mg/Al-LDH, thereby reducing the material's adsorption performance.^51^ In contrast, NO_3_^−^, Cl^−^, and Na^+^ exerted no significant effect on the indole adsorption process. HA also exhibited minimal interference with adsorption, likely because of the large size of HA molecules, which hinder their entry into the interior of the adsorbent.^52^

### 3.4 Comparative analysis of the performance with other adsorption materials

The performance of the Mg/Al-LDH@MOF composite prepared in this study was compared with several typical adsorbents reported in the literature (Fig. 4i). The results showed that the removal rate of indole by this composite was 9.26%, 1.14%, and 23.14% higher than those of ZSM-5,^53^ GAC,^54^ and AC,^55^ respectively. Although the removal rate of SP-FWBm^56^ was slightly higher than that of our material (by 1.23%), its dosage was as high as 500 times that of our adsorbent, leading to a significant increase in the practical application cost. In summary, the Mg/Al-LDH@MOF composite exhibits economic viability while maintaining high adsorption performance, making it a suitable and effective adsorbent for the removal of indole from wastewater.

### 3.5 Adsorption–desorption cycle regeneration performance

**Fig. 5:**
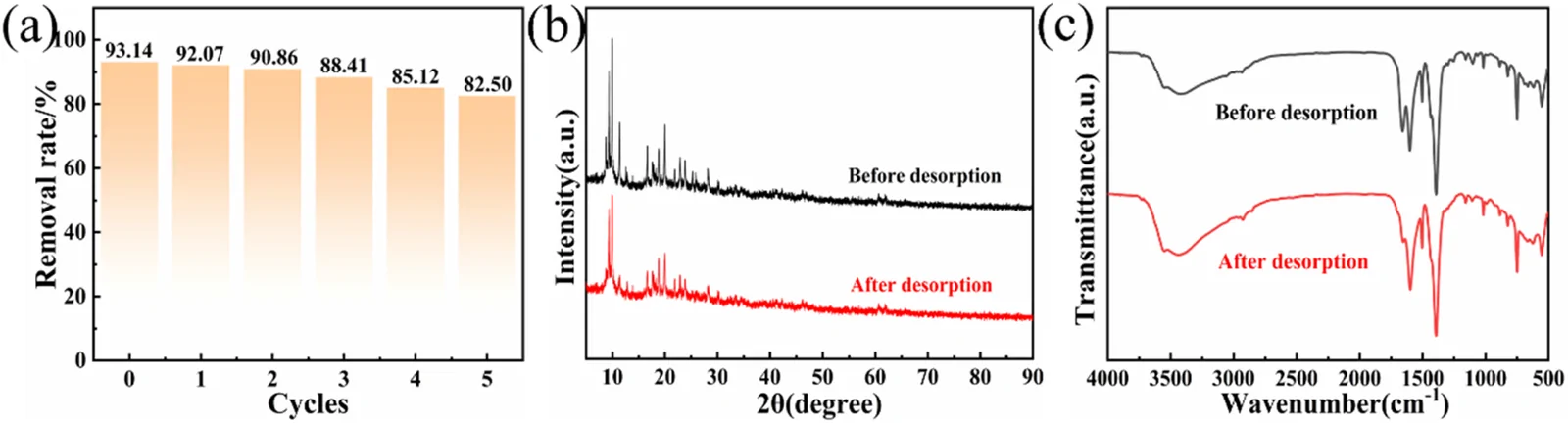
(a) Reusability of Mg/Al-LDH@MOF for indole removal. (b) XRD patterns and (c) FT-IR spectra of Mg/Al-LDH@MOF before and after desorption.

Under optimal reaction conditions, the Mg/Al-LDH@MIL-101(Fe) composite material, following pollutant adsorption, was centrifugally separated and recovered. It was subsequently washed with ethanol and dried for regeneration. Cycling tests (Fig. 5a) confirm exceptional stability, demonstrating excellent recyclability (>82% efficiency after 5 cycles). Furthermore, after five desorption cycles, the XRD (Fig. 5b) and FT-IR (Fig. 5c) spectra of the material exhibited no significant changes. This demonstrates that the material possesses excellent regeneration performance and recycling stability, thereby qualifying it as a reusable and efficient adsorbent.

### 3.6 Adsorption mechanism

**Fig. 6:**
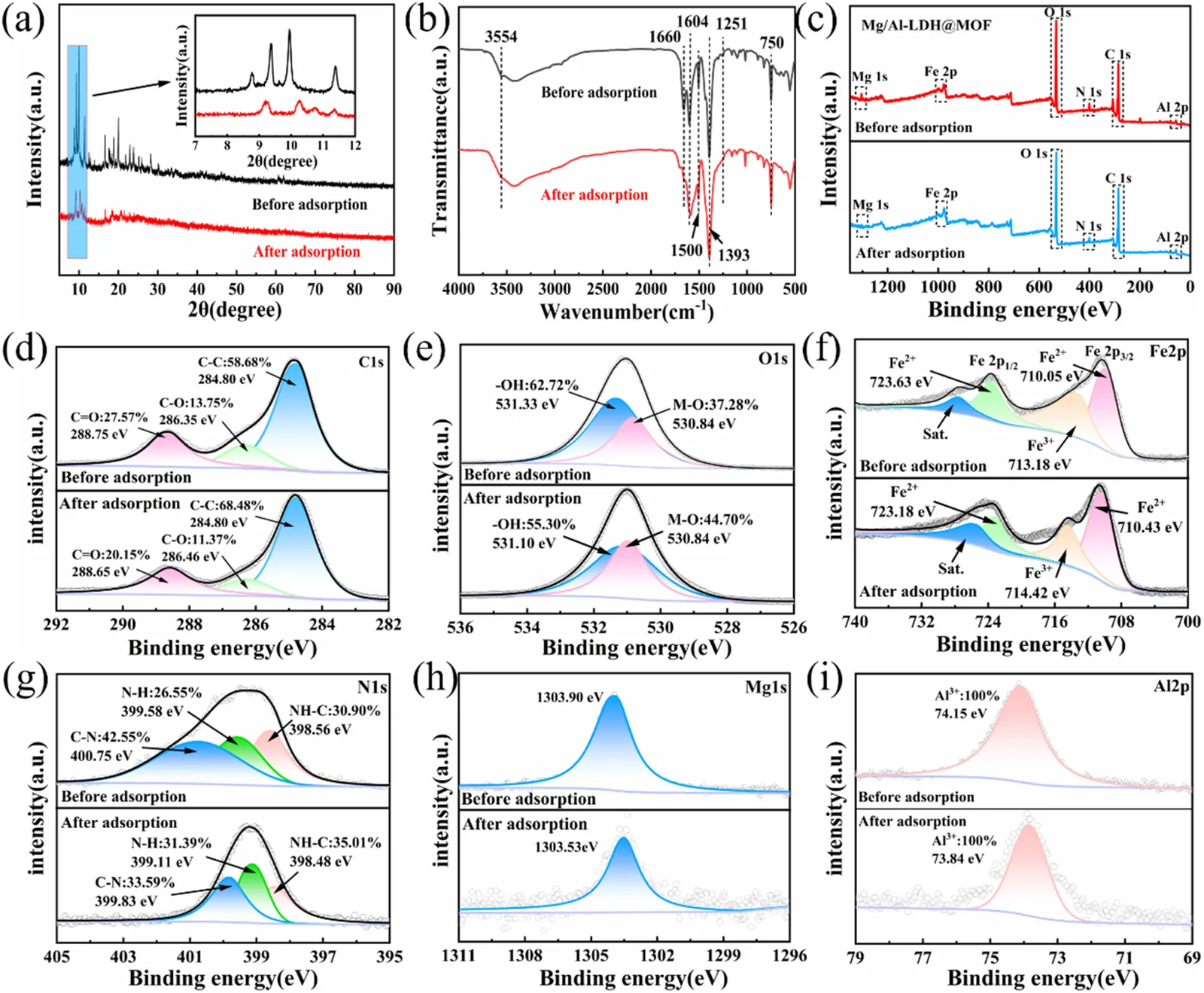
(a) XRD patterns, (b) FT-IR spectra, (c) XPS survey, and XPS spectra of (d) C 1s, (e) O 1s, (f) Fe 2p, (g) N 1s, (h) Mg 1s, and (i) Al 2p of Mg/Al-LDH@MOF before and after indole adsorption.

The XRD patterns of the composite before and after adsorption are presented in Fig. 6a. The characteristic diffraction peaks in the low-angle region (8–12°) exhibited shifts, which may be attributed to chemisorption.^57^Fig. 6b compares the FT-IR spectra of Mg/Al-LDH@MOF composites before after indole adsorption. Compared with the material before adsorption, the peak intensity at 1660 cm^−1^ of the material after adsorption is weakened, indicating the presence of π–π interactions between the composite material and indole.^58^ The characteristic peaks at 1251 cm^−1^, corresponding to the C–N bond, and 3554 cm^−1^, corresponding to the hydroxyl group, were weakened after adsorption, indicating that hydrogen bonds were formed between indole and the composite material.^59^ The slight shift of the 1604 cm^−1^ peak (assigned to the carboxyl group) suggests that the N–H group of indole interacts with the Mg/Al-LDH@MOF composite, possibly *via* hydrogen bonding or dipole–dipole interactions.^60^ FT-IR analysis demonstrated that the adsorption of indole by Mg/Al-LDH@MOF primarily occurs through hydrogen bonding and coordination between oxygen-containing functional groups on the surface and indole molecules.

Fig. 6c presents the XPS full spectrum of the adsorbent before and after use. Post-adsorption, the adsorbent retains characteristic peaks for C 1s, O 1s, Fe 2p, N 1s, Mg 1s, and Al 2p, with altered peak intensities, indicating the structural stability of the Mg/Al-LDH@MOF adsorbent. The increased ratio of C=C/C–C bonds following adsorption suggests enrichment of the indole aromatic structure on the material surface (Fig. 6d), confirming π–π interactions during the adsorption process (consistent with FT-IR characterization results). At the same time, the decreased ratio of C=O to C–O bonds indicates participation of oxygen-containing functional groups in adsorption, which formed a hydrogen bond network.^61^ The binding energies of the C=O and C–O bonds shifted from 288.75–286.35 eV to 288.65–286.46 eV, respectively. These shifts can be attributed to the formation of hydrogen bonding and π–π interactions between the adsorbent and indole during the adsorption process.^62^

According to previous studies, hydroxyl (–OH) can facilitate the adsorption process by generating protonated hydroxyl (–OH_2_^+^) species.^63^ The O 1s spectrum was further analyzed (Fig. 6e), revealing a decrease in the relative content of –OH from 62.72% to 55.30% before and after adsorption, respectively. This result confirms the participation of –OH groups in the adsorption process. The –OH peak shifted from 531.33 eV to 531.10 eV, further confirming that the hydroxyl groups act as hydrogen bond acceptors, forming hydrogen bonds with the N–H group of indole. Meanwhile, the –OH groups on the LDH layers can also immobilize indole molecules through hydrogen bonding.

Additionally, the intensity of the Fe 2p_3/2_ peak increased slightly after adsorption (Fig. 6f), which proved that the Fe–O groups could interact with indole *via* a metal surface complexation mechanism.^64^ XPS analysis of the N 1s region before and after adsorption (Fig. 6g) showed an increase in the relative proportions of N–H and NH–C bonds (to 31.39% and 35.01%, respectively) and a decrease in the C–N bond proportion (to 33.59%). This may be attributed to the enrichment of indole molecules on the material surface after adsorption, which enhances the characteristic signals of N–H and NH–C bonds (typical of indole). The C–N bond signal is partially obscured and forms hydrogen bonds with indole (consistent with FT-IR characterization results), leading to its reduced relative proportion. The binding energies of the NH–C, N–H, and C–N bonds decreased from 398.56 eV, 399.58 eV, and 400.75 eV to 398.48 eV, 399.11 eV, and 399.83 eV, respectively. This shift is attributed to the formation of hydrogen bonds between indole and the nitrogen sites, which facilitates electron cloud transfer toward the nitrogen sites, resulting in increased electron density.^65^Fig. 6h and i present the XPS spectra of Mg 1s and Al 2p before and after indole adsorption, respectively. Comparative analysis of the main peak positions (binding energies) of Mg 1s and Al 2p revealed that upon adsorption of indole by Mg/Al-LDH@MOF, the peak areas of Mg 1s and Al 2p decreased slightly, while their binding energies decreased from 1303.90 eV and 74.15 eV to 1303.53 eV and 73.84 eV, respectively. These changes suggest the potential formation of a metal bond bridge during the adsorption process.^66^

## 4. Conclusion

In summary, to systematically study the adsorption properties and mechanisms for the nitrogen-containing heterocyclic pollutant indole, we fabricated a structured Mg/Al-LDH@MIL-101(Fe) composites with the 3D hierarchical structure by *in situ* nucleation strategy. The resultant Mg/Al-LDH@MIL-101(Fe) exhibited outstanding indole adsorption capacities (175.732 mg g^−1^), which is significantly higher than that of the individual components (Mg/Al-LDH: 144.120 mg g^−1^; MIL-101(Fe): 102.563 mg g^−1^). The adsorption process involves a synergistic effect of electrostatic interactions, π–π stacking, hydrogen bonding, and metal coordination. Under optimized conditions (pH = 7, adsorbent dosage 0.1 g L^−1^, adsorption time 2 h, initial indole concentration 20 mg L^−1^), the indole removal rate by the composite material reached 93.14%. Furthermore, characterization techniques including XRD, FT-IR, Raman spectroscopy, and XPS provided direct evidence supporting the adsorption mechanism, confirming that indole can be effectively removed through electrostatic adsorption on the material surface, and π–π stacking. Importantly, the composite retained 82% of its capacity after five regeneration cycles and exhibited anti-interference capability, with an efficiency exceeding 86% in the presence of coexisting species. Thus, this work identifies Mg/Al-LDH@MIL-101(Fe) as a next-generation sorbent, capable of treating complex coking industrial wastewater while addressing global water sustainability challenges.

## Author contributions

Wei Zhang: conceptualization, validation, writing – original draft preparation, writing—review and editing, supervision, project administration, funding acquisition. Ding Peng: software, data curation, writing—review and editing, visualization. Aihe Wang: conceptualization, formal analysis, writing—review and editing, supervision. Hai Lin: methodology, resources, writing—review and editing, project administration. Xuchao Yan: methodology, resources, writing—review and editing, project administration. Zhihao Xu: software, investigation, writing—review and editing. Mingyou Yan: software, writing—review and editing, visualization. Zhenning Deng: conceptualization, validation, writing—review and editing.

## Conflicts of interest

There are no conflicts to declare.

## Supplementary Material

RA-016-D6RA04492A-s001

## Data Availability

The data that support the findings of this study are available upon request from the corresponding author. Supplementary information (SI): BET pore structure characteristics of the prepared materials; kinetic parameters for indole adsorption onto Mg/Al-LDH@MOF; fitting parameters of the Langmuir and Freundlich isotherm models for indole adsorption onto Mg/Al-LDH, MIL-101(Fe), and Mg/Al-LDH@MOF; thermodynamic parameters for indole adsorption onto Mg/Al-LDH@MIL-101(Fe) at different temperatures; SEM images (2 µm and 1 µm scales) of Mg/Al-LDH, MIL-101(Fe), and Mg/Al-LDH@MOF; high-resolution XPS spectra (C 1s, O 1s, N 1s) of Mg/Al-LDH@MOF; effect of adsorbent dosage on the removal of indole by Mg/Al-LDH, MIL-101(Fe), and Mg/Al-LDH@MOF. See DOI: https://doi.org/10.1039/d6ra04492a.
