# Supplementary material for: Efficient selective removal of indole by Mg/Al-LDH@MOF: mechanistic insights and adsorption behavior
Source: RSC Adv. 2026 Jul 31. Online ahead of print. doi: 10.1039/d6ra04492a (PMC13425732; doi:10.1039/d6ra04492a)
Supplement: RA-OLF-D6RA04492A-s001 [file RA-OLF-D6RA04492A-s001.pdf]

Supplementary Information (SI) for RSC Advances.
This journal is © The Royal Society of Chemistry 2026

Supplementary Information

Table S1. Pore structure characteristics of Mg/Al-LDH and Mg/Al-LDH@MOF.

	Mg/Al-LDH	MOF	Mg/Al-LDH@MOF
BET surface area（m^2/g）	12.204	82.63	146.384
BJH method adsorption surface area（m^2/g）	25.101	3.043	83.26
BJH method desorption surface area（m^2/g）	38.554	18.937	80.90
Micropore surface area（m^2/g）	-	48.136	109.661
Total pore volume e（cc/g）	0.028	0.013	0.102
BJH method adsorption pore volume（cc/g）	0.039	0.021	0.074
BJH method desorption pore volume（cc/g）	0.045	0.032	0.072
t-method micropore volume（cc/g）	-	0.018	0.057
SF method micropore volume（cc/g）	0.001	0.393	0.062
BJH method adsorption pore Diameter（nm）	1.188	6.278	1.189
BJH method desorption pore Diameter（nm）	1.348	1.344	1.35

Table S2. The adsorption kinetics of indole on Mg/Al-LDH@MOF and the fitting parameters of the Langmuir and the Freundlich isotherm adsorption models.

Models	Parameters	LDH/MIL-101(Fe)	LDH	MIL-101(Fe)
Pseudo-first order	$K_1(min^{-1})$	0.050	0.042	0.028
	$q_e(mg/g)$	39.05	34.22	31.23
	R^2	0.989	0.990	0.983
Pseudo-second order	$K_2(g/(mg{\cdot}min))$	0.002	0.002	0.001
	$q_e(mg/g)$	44.73	38.71	36.71
	R^2	0.999	0.997	0.991
Langmuir model	$q_m(mg/g)$	175.732	144.120	102.563
	$K_L(L/mg)$	0.058	0.037	0.033
	R^2	0.978	0.986	0.964
Freundlich model	$K_F(mg/g)/(mg/L)^n$	15.079	10.260	7.269
	n	1.676	1.626	1.821
	R^2	0.997	0.997	0.994

Table S3. Thermodynamic parameters for indole adsorption onto Mg/Al-LDH@MIL-101(Fe) at different temperatures.

T/K	ΔG^{θ}/(kJ·mol^{-1})	ΔH/(kJ·mol^{-1})	ΔS/(kJ·mol^{-1})	R^2
288	-0.33209	33.72674	118.0887	0.997
298	-1.41375			
308	-2.6984			

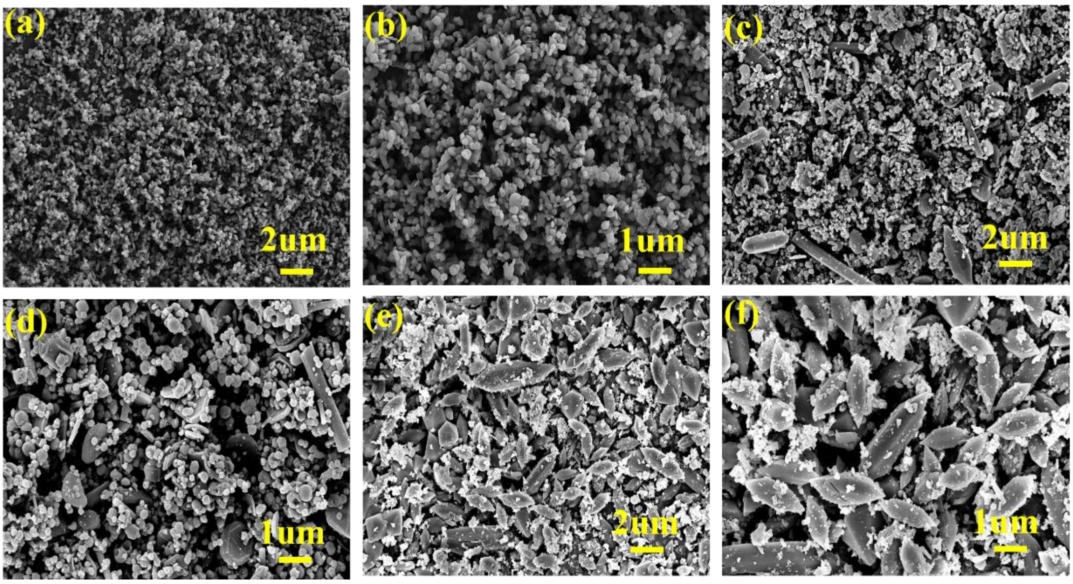

Fig. S1 SEM images of (a-b) Mg/Al-LDH, (c-d) MIL-101 (Fe), and (e-f) Mg/Al-LDH@MOF.

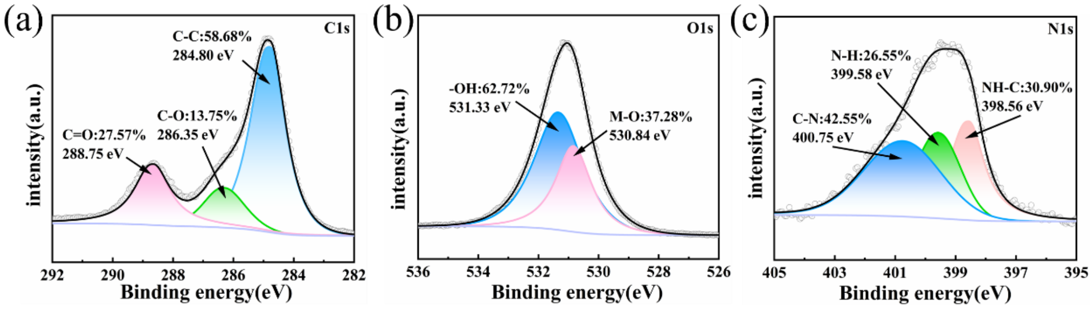

Fig. S2 XPS spectra of Mg/Al-LDH@MOF: (a) C 1s, (b) O 1s, and (c) N 1s.

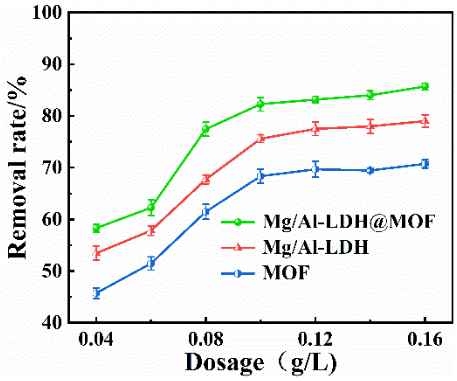

Fig. S3 Effects of adsorbent dosage on indole removal by Mg/Al-LDH, MOF, and Mg/Al-LDH@MOF (pH=7, adsorption time = 1h, concentration = 50mg/L, T = 25 ℃).
